# A Driver’s Physiology Sensor-Based Driving Risk Prediction Method for Lane-Changing Process Using Hidden Markov Model

**DOI:** 10.3390/s19122670

**Published:** 2019-06-13

**Authors:** Yan Li, Fan Wang, Hui Ke, Li-li Wang, Cheng-cheng Xu

**Affiliations:** 1 School of Highway, Chang’an University, Xi’an 710064, China; lyan@chd.edu.cn (Y.L.); wfssjj@chd.edu.cn (F.W.); answerkh@chd.edu.cn (H.K.); lery@chd.edu.cn (L.W.); 2 State Key Laboratory of Road Engineering Safety and Health in Cold and High-altitude Regions, CCCC First Highway Consultants Co., LTD, Xi’an 710075, China; 3 School of Transportation, Southeast University, Nanjing 211189, China

**Keywords:** driving risk prediction, hidden Markov model, physiology measurement sensor, vehicle dynamic data, lane changing

## Abstract

Lane changing is considered as one of the most dangerous driving behaviors because drivers have to deal with the traffic conflicts on both the current and target lanes. This study aimed to propose a method of predicting the driving risks during the lane-changing process using drivers’ physiology measurement data and vehicle dynamic data. All the data used in the proposed model were obtained by portable sensors with the capability of recording data in the actual driving process. A hidden Markov model (HMM) was proposed to link driving risk with drivers’ physiology information and vehicle dynamic data. The two-factor indicators were established to evaluate the performances of eye movement, heart rate variability, and vehicle dynamic parameters on driving risk. The standard deviation of normal to normal R–R intervals of the heart rate (SDNN), fixation duration, saccade range, and average speed were then selected as the input of the HMM. The HMM was trained and tested using field-observed data collected in Xi’an City. The proposed model using the data from the physiology measurement sensor can identify dangerous driving state from normal driving state and predict the transition probability between these two states. The results match the perceptions of the tested drivers with an accuracy rate of 90.67%. The proposed model can be used to develop proactive crash prevention strategies.

## 1. Introduction

### 1.1. Background

During the lane-changing process, drivers have to deal with complex traffic conflicts between current and target lanes, leading to increased driving workload [1]. If drivers acquire more information than their threshold workload, they may neglect some critical information related to safety. When drivers are aware of driving risks during the lane-changing process, they can wait for a more proper opportunity to process the lane-changing maneuver [2]. Accordingly, driving risk prediction during lane changing in real time plays a critical role in the proactive safety driving system [3]. The driving workload is the driver’s reaction to the driving demand, which is affected by the driver’s physiological state and traits, as well as the road environment [4]. Thus, the driver’s physiological status is one of the most critical factors in driving risk prediction models. However, most of the driving risk prediction models are generally based on vehicle dynamics [5], historical statistical data of incidents [6], or observed (natural) driving behaviors [7]. These models usually use the driver’s physiological information to represent the driver’s current natural status, rather than in the prediction of driving risk in the near future. The principal reason behind not using the driver’s physiological indicators in the driving risk prediction models might be that there is no direct connection between these indicators and the driving risk. In this way, we need to develop a model that can connect the driver’s physiology status with the driving risk for a driving task with a heavy workload, such as the lane-changing process. The proposed model should take full consideration of the complex interactive effects among driving behaviors, driver’s physiology status, vehicle dynamics, and surrounding traffic dynamics [8], whose measurement data are collected by multiple sensors in real time.

### 1.2. Literature Review

The influencing factors on driving tasks can be divided into the driver’s status and external environment factors [9]. Both physiology information and psychology information can be utilized to measure the driver’s status [10]. Physiology information mainly refers to the characteristics of driving actions. Inappropriate driving actions, or ever incorrect actions, such as insufficient/excessive braking/turning, is one the most critical inducements of traffic incidents [11]. If such inappropriate or incorrect actions can be identified timely, the safety conditions can be significantly improved by warning drivers about these dangerous actions [12]. Other physiology information, like individual characteristics and health conditions, can also have an impact on the driving actions [13]. Even though psychology conditions do not affect the driving actions directly, they can be used as indicators of the current physical status of the driver [14], which is because psychology conditions significantly affect judgment in the driving process. Physiological indicators, including the electroencephalogram (EEG) [15], electrocardiogram (ECG) [16], electromyography (EMG) [17], heart rate [18], eye gaze distribution [19], saccade range, blink frequency [20], and so on, were used to measure drivers’ physical status. Data were collected either in the field driving environment or on a driving simulator [21]. Collecting and processing the data in real time raises higher requirements on the sensors. The sensors must be sufficiently portable to minimize the impact on the participants and, meanwhile, they should also be powerful enough to process the data in real time [22]. The sensors utilized in the field driving environment should meet the requirements listed above. 

The data processing methods fall into three categories. The first category treats the normalized parameters of a specific driver as an eigenvector. The clustering methods [23] can then be utilized to classify these vectors into several categories. The driving risks of these categories can be identified by reviewing the original traffic dynamic status [24]. When the physiological data are continued in time, the second category of methods can be utilized. These methods will process the physiological data using the digital signal processing methods, like Fourier transform, spectrum analysis [25], wavelet transforms [26], and so on. With these technologies, the periodic characteristics of the selected parameter can be extracted, such as the non-sinus rhythm caused by unexpected traffic conditions. The third category of methods treat a specific parameter as a time series. They use historical data and data acquired in the last few time intervals to predict the driving risk in the coming time interval [27]. The driving risk prediction methods can be either a supervised learning process or a model-based prediction method. The supervised learning technologies include artificial neural network [28], Gaussian process mixture models [29], accident tree method [30], and support vector machine [31]. The samples of the driving risk are usually obtained by the Delphi method or judged by field observations [5]. The major drawback of the supervised learning method is that it cannot deal with the conditions that are not contained in the training set. Moreover, the evaluation of the driving risk may be subjective. The model-based prediction tries to establish a connection between observed parameters and driving risk. Although existing studies proposed a number of models [32] to predict the driver’s status using the physiological indicators, additional work is still needed to analyze the interactions among the influence factors with complicated driving tasks, such as the lane-changing process [33]. 

Existing literature shows that the physiological indicators can be utilized to describe the driver’s status in a specific driving environment using various sensors. The driving risk is a comprehensive result of the driver’s physiological status, vehicle dynamic data, and external environment. Although single or several influence factors were considered to represent the driver’s status, little research focuses on the prediction on driving risk with the considerations of all the factors. These gaps are summarized as follows: ① the existing prediction models mainly based on one type of performance measurement factor, such as the Time-To-Collision (TTC) extracted from the surveillance video or the driver’s physiological indicators (such as the driving fatigue degree). A driving risk prediction model is required with the consideration of both vehicle dynamic and driver’s physiological information; ② the prediction models should have the capability of extracting the potential connections between the driving risk and the performance indicators of the driver’s physiology and vehicle dynamic status. 

### 1.3. Objective and Contributions

The objective of this study was to develop a compound model for the prediction of the driving risk during one of the most dangerous driving stages, the lane-changing process, with the consideration of both the driver’s physiological information and vehicle dynamic information. For the purpose of collecting comprehensive data for driving risk prediction during the lane-changing process, a field experiment was designed to collect the driver’s eye movement information, an electrocardiogram, and vehicle dynamic information. With the description using the two-factor indicator for processed data, a hidden Markov Model (HMM) was used to link the experimental performance indicators with the driving risk during the lane-changing process.

The major contributions of this study are twofold: (1) the driver’s physiological information is added along with the vehicle dynamic information in the driving risk prediction model during the lane-changing process. All the major influence factors are considered in the driving risk prediction model; (2) the potential connections between the major influence factors and driving risk are linked using the hidden Markov model, which can predict the driving risk using the state transmit probabilities.

The remainder of this paper is organized as follows: Section 2 shows the way to select effective sensors to collect full and accurate data in the field experiment for further research. A two-factor indicator is proposed to describe the driver’s physiology and vehicle dynamic status in Section 3. Section 4 establishes a hidden Markov model-based driving risk prediction model. Section 5 demonstrates the experimental results and discusses the results. Section 6 concludes the paper.

## 2. Experiment

### 2.1. Experiment Design

**Figure 1:**
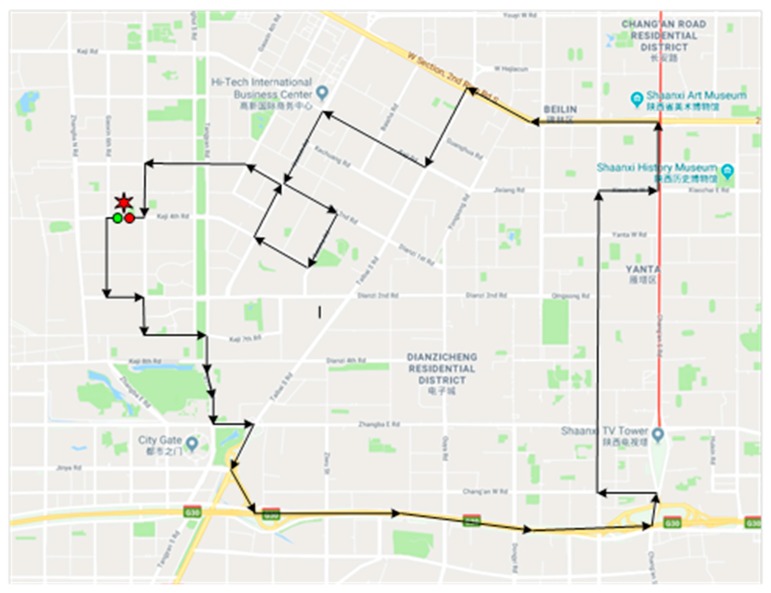
Selected experimental route.

The field experiment method was selected to collect the driver’s physiology data and vehicle dynamic data for the purpose of estimating the driving risk during the lane-changing process. Data used in this study were collected in Xi’an City. As shown in Figure 1, the total length of the tested route was 26.6 km. The selected experimental route included urban freeways, arterials, and collectors, as well as intersections and interchanges. The speed limits of the urban freeways, arterials, and collectors were 70 km/h, 60 km/h, and 50 km/h, respectively. In total, 25 drivers were selected to finish the experimental route. All of them were healthy and had a driving age longer than three years, with an average number of kilometers traveled per year of more than 6000 km. Eight drivers were female. The experiments were carried out during peak hours (7:00–10:00 a.m., 3:00–6:00 p.m.) on eight sunny workdays. All the participants were asked to rest well the previous night. Tobacco, wine, tea, coffee, and any other food or drug that might affect the driver’s physiology indicators were forbidden on the previous day. Before the formal test, the participants were asked to take a 10-min test drive outside the driveway to familiarize them with the experimental vehicle and devices. The drivers’ physiological data (including the eye movement data and electrophysiology data) and vehicle dynamic data were collected as the input dataset of the driving risk prediction model. The eye movement information included eye fixation, saccade duration, blink frequency, pupil diameter, and field of front vision. The drivers’ electrophysiology data included an ECG and EMG. Meanwhile, we also collected the breath rate, oxyhemoglobin saturation degree, and blood pressure. The vehicle dynamic indicators included speed, acceleration/deceleration, real-time vehicle position, and the status of the various vehicle subsystems.

### 2.2. Equipment Selection and Installations

**Figure 2:**
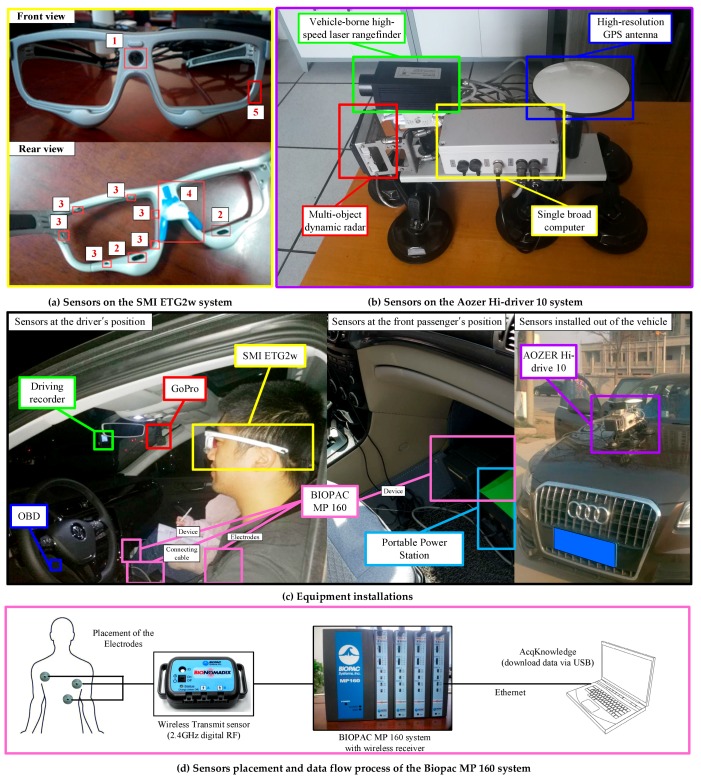
Equipment installation and sensors on the equipment.

Both the diver’s physiological data and vehicle dynamic data are critical to our research. In order to improve the accuracy of the data, we chose some commercial sensors, which will be more reliable in the experiment process. The BIOPAC MP 160 physiological data acquisition system, SMI ETG 2w eye-tracking glasses, AOZER Hi-Drive 10 vehicle dynamic data acquisition system, and the OBD (on-board diagnostics) were selected in the experiment. The equipment installations on the test vehicle are illustrated in Figure 2.

The BIOPAC MP 160 physiological data acquisition system is a flexible, proven modular data acquisition and analysis system for life science research. This system has 16 channels with the capabilities of recording reproducible ECG, EEG, EMG, blood pressure, and more data. Each kind of data can be measured by an independent detachable module. The ECG data can represent both basic physiological characteristics and driver reactions to real-time incidents with adequate robustness. Thus, we selected the ECG data as the measurement indicator for the purpose of minimizing the influence on the drivers. The MP 160 system uses micro-electrodes to record the ECG data with the sampling rate at a speed of 400 kHz (aggregated). To measure the heart’s electrical activity accurately, proper electrode placement is crucial. Three electrodes are required to be pasted on the chest by the MP 160 system. The placement of the electrodes is shown in Figure 2d. The MP 160 system uses an Ethernet cable to send the recorded signal to a laptop with AcqKnowledge software. Multiple modules of the MP160 system can easily interface with the one laptop with AcqKnowledge software. 

The eye movement information was collected using the SMI Eye-Tracking Glasses 2 Wireless (SMI ETG 2w) system. The SMI ETG 2w system can record a driver’s natural gaze behavior in real time with adequate robustness, mobility, and ease of use. As shown in Figure 2a, there was a high-resolution scene camera (number 1 in Figure 2a) in the middle of the front view of the glass, and one microphone on the left side (number 5 in Figure 2a). The lens could either use the non-shaded lens or assorted myopia lens. Two high-speed eye cameras with the technology of infrared detection (number 2 in Figure 2a) were located at the rear side bottom of the glass, facing the pupil. The eye cameras can track native, binocular eye movement information over the whole trackable field of view with a sample rate of 120 Hz. Twelve fill-in light-emitting diode (LED) lights (number 3 in Figure 2a) at the rear side of the glass worked constantly to ensure the accuracy of the sensing process. The nose rest (number 4 in Figure 2a is the slot of the nose rest) was also exchangeable to provide comfortable wear experience during the experiment. The SMI ETG 2w system uses a lightweight recorder based on an Android smartphone. The operators can control and calibrate the glass, as well as collect and view real-time participant properties in the smartphone-based recorder or laptop. For enhanced productivity, annotations can be used in SMI BeGaze software to efficiently focus data analysis on relevant sequences of the recorded data.

The vehicle dynamic information, including the real-time speed, position, and gap between the leading vehicles, was collected by the Aozer Hi-Drive 10 system (Figure 2b). The Hi-Drive system contains a multi-object dynamic radar sensor, a vehicle-borne high-speed laser rangefinder sensor, and a high-resolution global positioning system (GPS) sensor. The data collected from these sensors were recorded by the single broad computer connected to these sensors and then transmitted to the laptop. The position and related speed of the tested vehicle were recorded by the high-resolution GPS sensor. The gaps between the tested vehicle and other leading vehicles could be collected by the multi-object dynamic radar sensor and the vehicle-borne high-speed laser rangefinder sensor. The laser rangefinder sensor has greater accuracy, but a narrower detection range. In this way, the data of the laser rangefinder sensor were selected as the verification of dynamic radar. In addition to the gaps, the dynamic radar sensor could also obtain the accelerations and speeds of the leading vehicles. The laser rangefinder data become the major data source when the dynamic radar does not work well, such as in low-speed situations. We also collected the engine speed and speed using the OBD for further verification of the driving process. As shown in Figure 2c, the Hi-Drive system was installed on the hood of the tested vehicle.

### 2.3. Supplemental Illustrations of the Experiments

Even with the support of these commercial sensors, there were still some failures during the experiment. We had to park the vehicle to the roadside and re-calibrated the experimental equipment, which had a strong influence on the drivers. Meanwhile, one of the most important points of the field experiment is that we should minimize the influences of the equipment on the drivers. Although a training section and many measures (such as wearing the contact lenses) were carried out, there were still certain influences on the drivers. The indicators of the first ten to fifteen minutes usually had significant volatility. In this way, these data were removed to ensure the data utilized in this research can represent the drivers’ characteristics. 

In our experiments, if the drivers wore the sweater, which may discharge static electricity, the ECG data would have lots of noise. These data were carefully processed manually and used as a reference. Loose clothes are recommended in the experiment that collects ECG data. Meantime, portable devices with the capabilities of collecting heart rate, such as heart rate belt, are potential devices for this experiment, which can be utilized along with the ECG recording devices.

## 3. Modeling Driving Risk Using Driver’s Physiology Information

### 3.1. Driving Risk during Lane-Changing Process

According to the characteristics of the overtaking process, it can be divided into four stages: car following, perception, intention, and execution [19]. During the car-following stage, the vehicle runs smoothly. At this time, drivers mainly focus on the front view of the vehicle. The drivers need to measure the gap between the current vehicle to the leading and following vehicles in both the current lane and target lane. Meantime, they should also make the judgment about the consequences of possible actions. The drivers should continually repeat previous judgments with regard to the related vehicle dynamic during the execution stage until they change the vehicle to the target lane. Because the average speed of the original lane should be less than the target lane, there will be less risk if the studied vehicle decides to return to the original lane after the overtaking process.

The lane changing action usually requires higher driving skills than other driving tasks, such as car following. The drivers should deal with the interactions between the front and rear vehicles in both the current lane and target lane during the lane-changing process. The drivers should maintain the vehicle speed while judging the real-time gaps between the leading vehicles and rear vehicles. Because the driver focuses on the lane-changing task, they may miss other turbulences, which will raise the driving risk. In this way, the driving risk in heavy-flow conditions will be higher. Based on the characteristics of the lane-changing process and the incentives of driving risk, the levels of service and actions of the lane-changing process are the critical influence factors on the driving risk.

### 3.2. Two-Factor Indicators on Driving Risk

Two-factor indicators were proposed to evaluate the performances of selected indicators on driving risk. The two-factor indicators should be capable of evaluating the probability of the events and the differences between the selected two factors. In this way, the ratio between the variances of two sets of sample data can indicate the degree of deviation of the samples. The indicator *I*_1_ can be calculated using Equation (1). If *I*_1_ is greater than the threshold *I*_t_, the samples affected by the selected influence factor are significantly different, and vice versa.

(1) $$I_{1}=\frac{S_{i}^{2}}{S_{j}^{2}}=\sum \frac{{\left(x_{im}-\bar{x_{i}}\right)}^{2}}{m-1}/\sum \frac{{\left(x_{jn}-\bar{x_{j}}\right)}^{2}}{n-1},$$

where *S_i_* and *S_j_* are the variances of sample datasets *i* and *j*; there are *m* and *n* elements in sample datasets *i* and *j*, and the samples can be expressed as *x_im_* and *x_jn_*.

The indicator *I*_2_ can represent the probability caused by sampling error if a specific measure is applied. This indicator can reflect the overall influence of the sampling error. If the value of *I*_2_ is less than the pre-set significance level α, we can reject the hypothesis, i.e., there are significant differences between the sets of sample data. Based on the significance test method, there will be some influence if *I*_2_ is less than 0.05, whereas the influence is significant when *I*_2_ is less than 0.01, and the influence is extremely significant when *I*_2_ is less than 0.001.

As we summarized in the literature review section, the driving risk is mainly affected by the driver’s physiological status and external roadway environment. The driver’s workload will be significantly different under different levels of service (LOS), which makes it the most critical influence factor among the external roadway environment factors [34]. When the traffic demand is under the road capacity, the workload will increase as the traffic demand grows. However, for the purposed of seeking for a better driving environment, the driver might try to change lane frequently under oversaturated condition, which may cause a different workload. Fatigue is one of the most critical influence factors for driving risk on motorways or major roadways, and it is mainly caused by long driving duration [35]. Thus, the LOS and driving duration are reasonable classification criteria for the roadway environment and driver’s physiological status, respectively [36]. 

The Highway Capacity Manual (HCM) divides the LOS into six levels: LOS A as the free flow, LOS B as the reasonably free flow, LOS C as the stable flow, LOS D as the approaching unstable flow, LOS E as the unstable flow, and LOS F as the forced or breakdown flow. To reduce the complexity of analysis, we combined the LOS with a similar pattern of the indicators into free flow (LOS I, corresponding to LOS A and B in the HCM), stable flow (LOS II, corresponding to LOS C and D in the HCM), unstable flow (LOS III or LOS E in the HCM), and forced or breakdown flow (LOS IV or LOS F in the HCM). Literature shows that the safe limit for monotonous highway driving is 80 min [35] and the drivers should take a break every 120 min [37]. Thus, we classified the driving duration into four groups: less than 40 min, 40–80 min, 80–120 min, and greater than 120 min. 

To evaluate the influence of selected factors on the driving risk, one-way ANOVA (analysis of variance) for the influence factors of each overtaking stage with related driving duration interval and LOS was processed. When the indicator *I*_2_ is less than the pre-set significance level, the variance obtained from the ANOVA method can be utilized to calculate the indicator *I*_1_. The evaluation of indicator *I*_1_ should be analyzed based on the characteristics of the selected indicator. 

### 3.3. Evaluations of Eye Movement Factors

The fixation duration, saccade range, and variability of pupil diameter, which were obtained from the SMI ETG 2w eye-tracking glasses, were selected as the influence factors on driving risk. The fixation duration is the time of maintaining visual gaze on a single location. In medicine, the fixation duration represents the difficulty of capturing and processing visual information by the driver, which indirectly reflects driver fatigue. The saccade is a quick, simultaneous movement of both eyes between two or more phases of fixation in the same direction. In the driving process, the saccade range represents a driver’s visual angle. The driver is considered as being nervous when he/she has a large saccade range, which will lead to poor recognition of the road environment [38]. The diameter of the pupil controls the amount of light entering the driver’s eye, which may have a high correlation with driver fatigue [39]. However, it may also be affected by emotion or lighting. 

**Figure 3:**
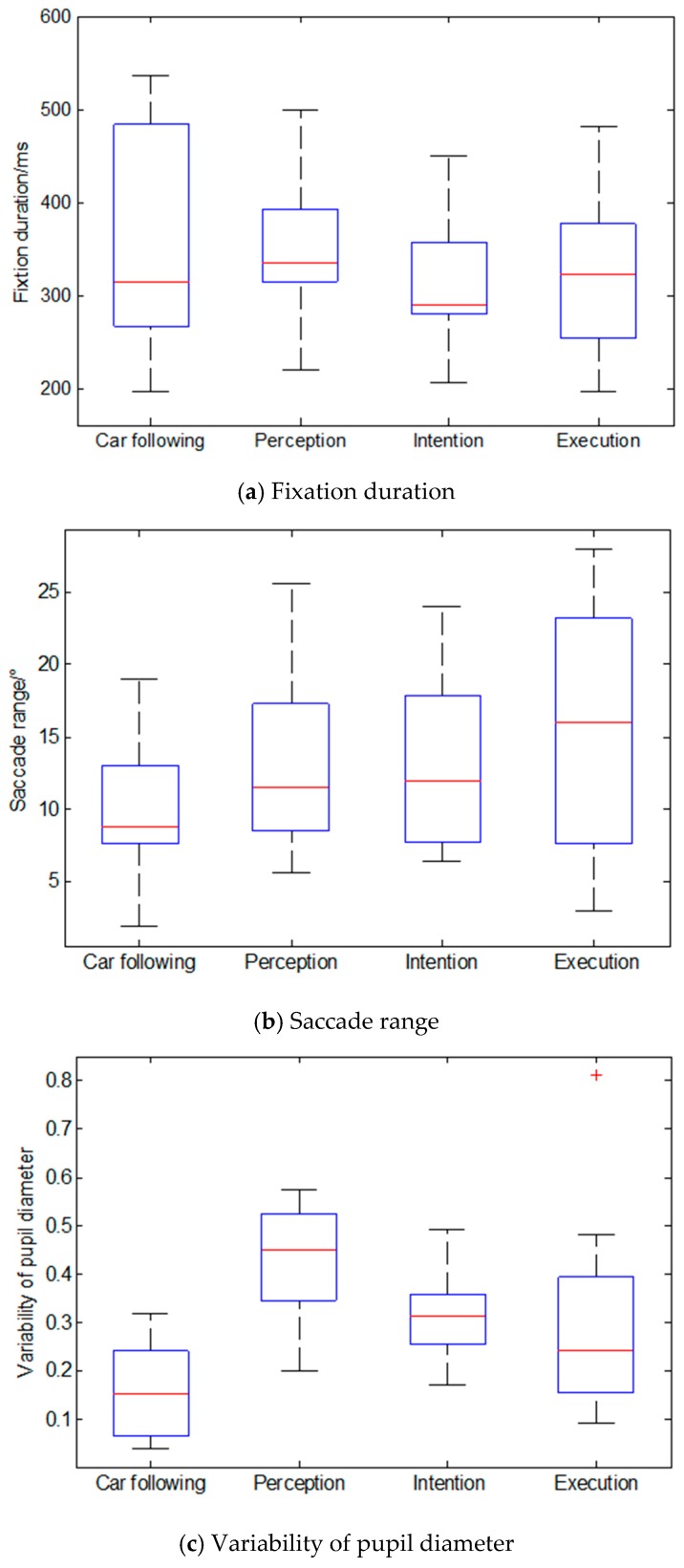
Characteristics of eye movement parameters of the lane-changing process.

The measurements of the selected parameters in each lane-changing stage are summarized in Figure 3. The results illustrate that the drivers have the most active eye movement in the intention stage and execution stage. The eye movement characteristics in the perception stage show a similar pattern in the car-following stage. The two-factor indicators for the LOS and driving duration during all lane-changing process were calculated, as shown in Table 1. Table 1 indicates that the fixation duration and saccade range had a significant influence on the level of service and driving duration during the intention and execution stages. The variability of pupil diameter had a certain influence on the level of service during the intention and execution stages, while it had no influence on driving duration. The eye movement factors indicated that there was no other influence during the lane-changing process. Based on the evaluations of eye movement factors, the fixation duration and saccade range were selected as the inputs of the driving risk prediction model.

**Figure 4:**
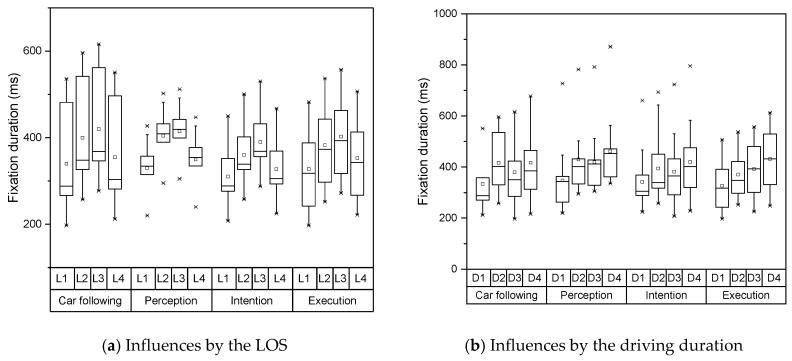
Distribution of fixation duration under various conditions. LOS—level of service.

**Figure 5:**
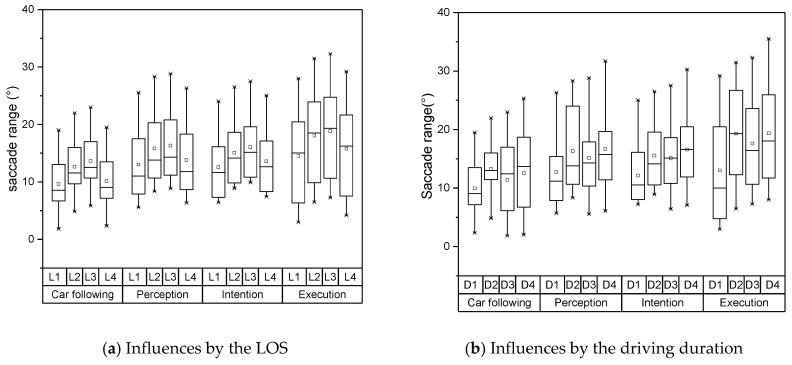
Distribution of saccade range under various conditions.

The distributions of these two indicators under each LOS and driving duration are shown in Figure 4 and Figure 5. Both fixation duration and saccade range represented disparate patterns under various LOS and driving duration. The fixation durations had large variances in both car following stage and execution stage, which demonstrated the drivers had limited information to observe. In contrary, the drivers needed to obtain more visual information during the perception stage and intention stage. They only spent little time on a single object. As the LOS increased under the unsaturated conditions, the fixation duration increased continuously. This means the drivers need longer fixation duration and large saccade range to extract effective information when external environmental information grows. Under the saturated condition, the tested vehicles constantly searched the chances to change lanes, which lead to the decline of fixation duration. Meantime, for the reason that there were fewer choices for the drivers, they represented a smaller saccade range under the saturated condition. With the increase of driving duration, both indicators and their variances showed a trend of increasing continuously. Both indicators showed different features at the interval of 40-80 mins. Based on the literature [35], the driving duration of 80 minutes is the safe limit for monotonous highway driving, which may explain this extraordinary feature.

### 3.4. Evaluations of ECG Factors

Heart rate variability (HRV) is the fluctuation in the time interval between adjacent heartbeats [40]. The heart rate variability can index the driver’s neuro-cardiac function during the lane-changing process. The HRV metrics can be described using both the time domain and frequency domain, which can be extracted from the electrocardiogram (ECG) morphology. The LF/HF (ratio of absolute power of the low-frequency band (LF) to that of the high-frequency band (HF), an HRV frequency-domain measure), the standard deviation of normal to normal R–R intervals of the heart rate (SDNN, an HRV time-domain measure), and coefficient of variation of the R–R intervals (CV, an HRV time-domain measure), which were obtained from the BIOPAC MP 160 system, were selected as the influence factors on driving risk. In medicine, larger time-domain metrics indicate a vigorous cardiopulmonary function, which means that the human body can adjust the amount of oxygen being transmitted to the organs by changing the heart rate based on the events happening during the driving process. The frequency-domain indicator LF represents the characteristics of the sympathetic nervous system, which has long-term effects on the driver’s physiological indicators related to cardiopulmonary function, while the HF represents the characteristics of the parasympathetic nervous system, which controls the driver’s reactions to incidents during the driving process. The LF increases and the HF decreases when the driving duration increases, i.e., the driver is transitioning to a fatigued state. 

**Figure 6:**
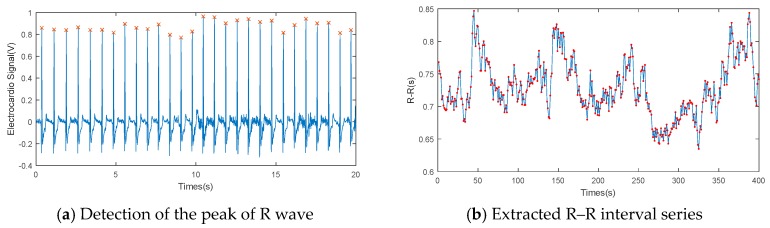
Electrocardiogram (ECG) data processing.

**Figure 7:**
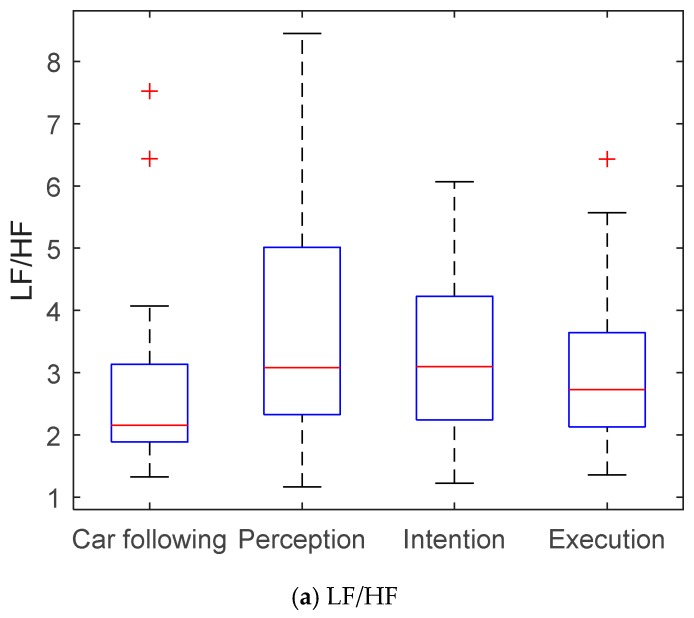

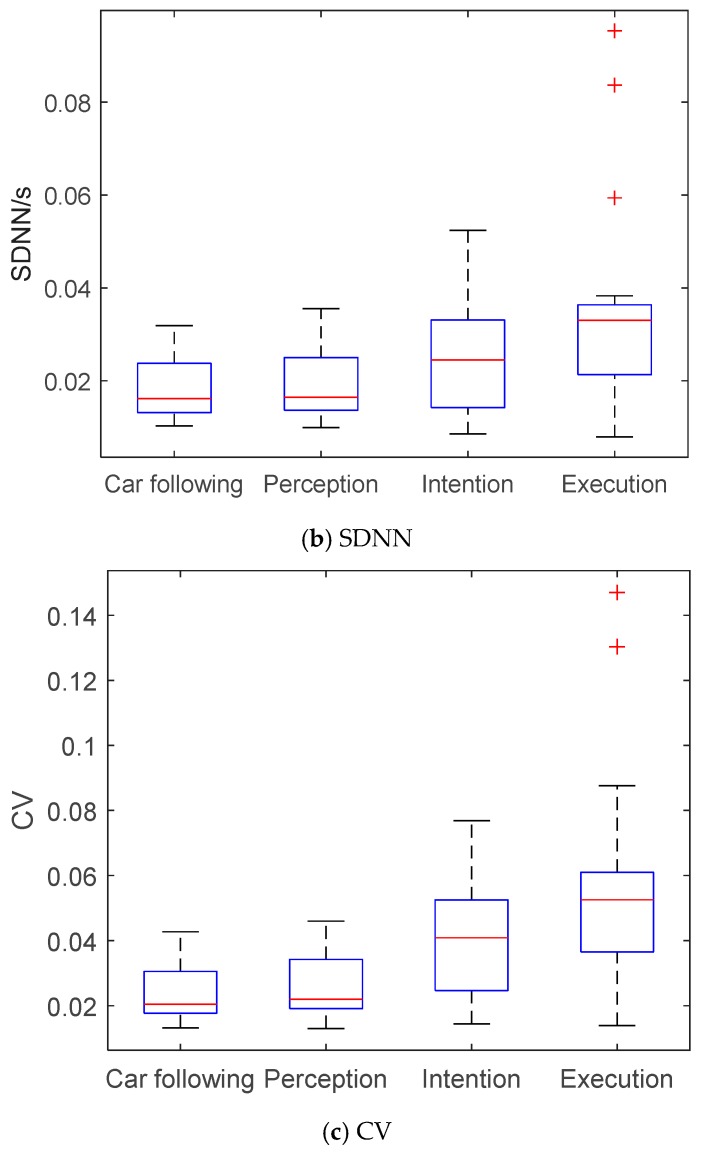
Characteristics of HRV parameters of lane changing process. LF/HF—ratio of absolute power of the low-frequency band to that of the high-frequency band; SDNN—standard deviation of normal to normal R–R intervals of the heart rate; CV—coefficient of variation of the R–R intervals.

The ECG signal was extracted from AcqKnowledge software. As shown in Figure 6, the R wave peaks in the ECG signal were detected to obtain the time series of the R–R interval. Both LF power and HF power can be calculated using Welch’s method [41]. The SDNN and CV can be calculated directly using the R–R intervals during the lane-changing process. Unlike the eye movement characteristics, statistics of each parameter shown in Figure 7 indicate that the HRV parameters in both the perception stage and intention stage had similar patterns. Thus, the drivers were shown to be active in mind from the perception stage. However, their actions became active from the latter intention stage. Table 2 shows the influence of ECG factors on driving risk during the lane-changing process. Almost all the ECG indicators showed strong influences on the level of service during all lane-changing stages. However, the SDNN and CV represented a significant influence on driving duration, while the LF/HF showed very little influence. Based on the evaluations of ECG factors, the SDNN was selected as the input of the driving risk prediction model. 

**Figure 8:**
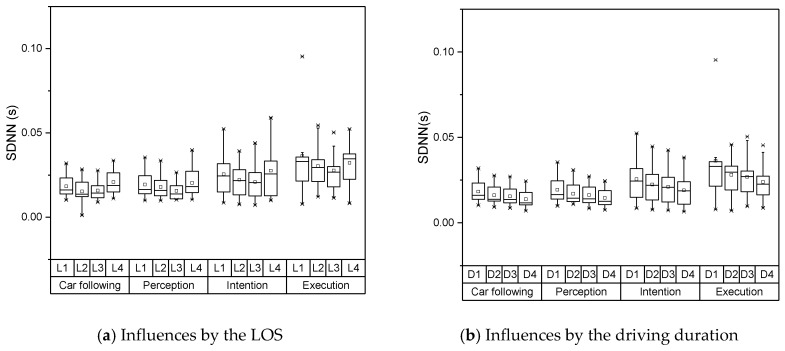
Distribution of SDNN under various conditions.

The distributions of SDNN under each LOS and driving duration are shown in Figure 8. The distributions of SDNN under each LOS and driving duration are shown in Figure 8. The SDNN in the car following stage and perception stage represented a relatively stable pattern, which meant the workload of the limb movement didn’t increase. The drivers began to be nerves and have certain physical movements when the lane changing process was turned into the implementation process. At this time, the driver’s heart rate might have greater variabilities. Similarly, the SDNN continued to decline when the LOS increases under the unsaturated state. This characteristic may relate to driving speed. With a lower LOS, the drivers would like to choose a high speed, which needs them to maintain an excited state. The drivers might become excited again when they decide to change lanes continually under the oversaturated state. As the driving fatigue will accumulate continuously as the driving duration increase, the SDNN of each lane changing stage will decrease respectively.

### 3.5. Evaluations of Vehicle Dynamic Factors

**Figure 9:**
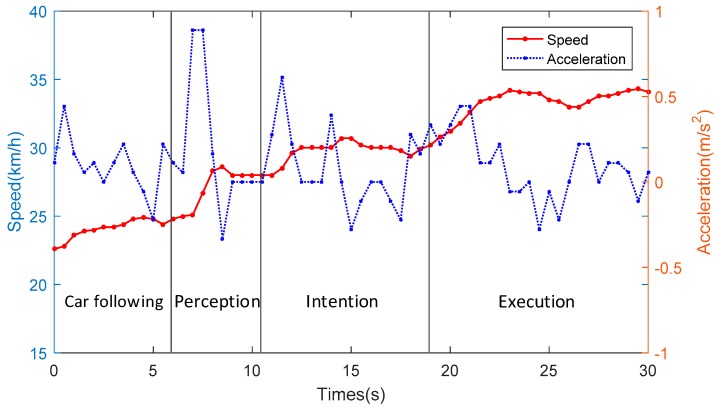
Variations of vehicle dynamic parameters of one specific lane-changing process.

**Figure 10:**
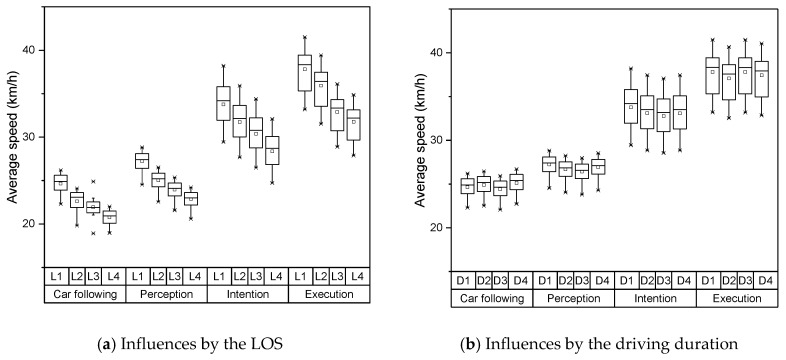
Distribution of average speed under various conditions.

The average vehicle speed and acceleration, which were obtained from the AOZER Hi-Drive 10 vehicle dynamic recording system, were selected as the influence factors on driving risk in the field of vehicle dynamics. The variations of the vehicle speed and acceleration during one specific lane-changing process are shown in Figure 9. The speed and acceleration data indicate that the drivers implemented operations on their vehicles only from the execution stage. As shown in Table 3, the two-factor indicators during all lane-changing processes were calculated. It can be judged from Table 3 that the average speeds of all events were significantly affected in the fields of the level of service and driving duration. The acceleration factors had a strong influence on the level of service, and almost had no effect on driving duration. Thus, the average speed was selected as the input of the driving risk prediction model. The distributions of average speed under each LOS and driving duration are shown in Figure 10.

## 4. Hidden Markov Model-Based Driving Risk Prediction

### 4.1. Driving Process and Hidden Markov Model

The hidden Markov model (HMM) assumes that the modeling system is a Markov process. However, the states of the Markov process cannot be observed, i.e., they represent hidden states. Some variables that are related to the hidden states can be observed with no Markov characteristics. In this way, we can estimate the Markov transition process of the HMM using the observed data. 

**Figure 11:**
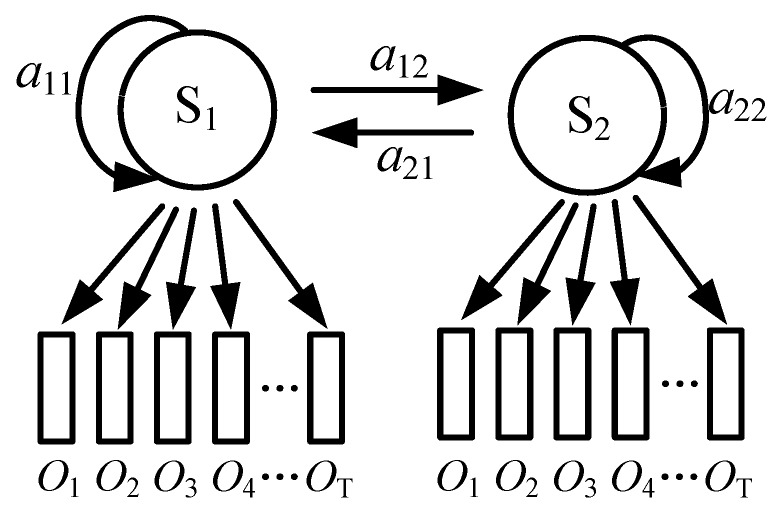
Structure of the state transition probability matrix.

The structure of the HMM can be represented by Equation (2). The driving process is a continuous time-dependent process, whose state can be transferred to the next state or can stay with the current state. In this way, the left to right model was selected to model the state transition probability matrix ***A***, whose structure is shown in Figure 11. The element of A can be calculated using Equation (3). The initial probability distribution ***Π*** can be either 1 (*i* = 1) or 0 (*i* ≠ 1).

(2) λ=(A,B,Π),

where ***B*** is the probability density function of the outputs.

(3) $$a_{ij}\{\begin{array}{cc} \neq 0 & \begin{array}{ccc} j=i & or & j=i+1 \end{array}\\ =1 & i=j=N\\ =0 & others \end{array}.$$

### 4.2. HMM-Based Driving Risk Prediction

The observed eye movement, ECG, and vehicle dynamic parameters were selected as the inputs to predict the driving risk during the lane-changing process. Based on the influence of these parameters on the driving risk, the fixation duration, saccade range, SDNN, and average speed were selected as the observed inputs. The prediction result can be represented as the driving state being safe or dangerous. 

The initial settings of the HMM are listed as follows: the number of hidden states *N* was two (safe or dangerous); the number of observable parameters *M* was 4; the initial state transition probability matrix ***A*^0^** could be obtained by the averaging method shown in Equation (4); the observed probability matrix ***B_k_*** could be calculated from the *k*th observation parameters; the initial probability distribution of the *l*th driver ***Π_l_*** could be obtained by the averaging method in Equation (5).

(4) $$A_{}^{0}=\left[\begin{array}{cc} 0.5 & 0.5\\ 0.5 & 0.5 \end{array}\right];$$

(5) $$\Pi_{l}^{0}=\left[\begin{array}{cc} 0.5 & 0.5 \end{array}\right].$$

The HMM-based driving risk prediction can be accomplished using the following procedure:

Step 1. Convert the state transition probability matrix into a random matrix using the mixed weight vector ***C_jm_***. The vector of the mean values ***μ_jm_*** and covariance matrix ***U_jm_*** should be the mean value and covariance of the entire sample (*M* = 1) or part of the sample (*M* ≥ 2).
Step 2. Calculate the forward frequency *α*_t_(*i*) and backward probability *β_i_*(*j*) using the current sample set.
Step 3. Calculate the probability of auxiliary variable *ε_i_*(*j*, *m*) and estimate the value of $\hat{\lambda }$ using the equation $\hat{\lambda }=(\Pi ,A,C_{jm},\mu_{jm},U_{jm})$.
Step 4. Calculate the likelihood probability $P(O/\hat{\lambda })$.
  Step 5. If the $P(O/\hat{\lambda })$ is increased, the sample should be re-estimated using the estimation of $\hat{\lambda }$ in Step 3. Otherwise, the algorithm should return to Step 2 until $P(O/\hat{\lambda })$ does not increase anymore. At this time, the value of the parameter $\hat{\lambda }$ is the output of the algorithm.

## 5. Experiment Results

The proposed model was tested using MATLAB software. In total, 500 sets of data samples of the physiological parameters during the lane-changing process were collected. The drivers were asked to review the video of their driving events. Then, they were asked to evaluate the degree of safety of their actions. The HMM was trained with 425 samples. The rest samples were selected to evaluate the performance of the prediction.

(6) $$\{\begin{array}{c} \Pi =\left[\begin{array}{cc} 0.8584 & 0.1416 \end{array}\right]\\ \hat{A}=\left[\begin{array}{cc} 0.7529 & 0.2471\\ 0.2537 & 0.7463 \end{array}\right]\\ \hat{B}=\left[\begin{array}{cccc} 0.1369 & 0.2536 & 0.4359 & 0.1736\\ 0.3726 & 0.2108 & 0.1903 & 0.2263 \end{array}\right] \end{array}$$

The training results are shown in Equation (6). The results indicate that 85.84% of driving actions during the lane-changing process were deemed safe and the rest of them were deemed dangerous. The probability of keeping a safe driving state was 75.29%, and the probability of continuing a dangerous state was 74.63%. The probability of transferring a safe state to a dangerous state was 24.71%, and the opposite probability was 25.37%. The emission probabilities of the safe states were 13.69% of the fixation duration, 25.36% of the saccade range, 43.59% of the SDNN, and 17.36% of the average speed. Meantime, the emission probabilities of the dangerous states were 37.26% of the fixation duration, 21.08% of the saccade range, 19.03% of the SDNN, and 22.63% of the average speed.

We found that 68 out of 75 predictions successfully matched the evaluations of the drivers. In this way, the accuracy rate of the prediction was 90.67%. When we reviewed the four events with divergences between our prediction and the driver’s perception, we found that it was difficult to distinguish the driving risk of these events. Most driving actions fell into an intermediate range between safe driving and aggressive driving. In this way, the proposed model can predict the driving risk with high accuracy.

In order to evaluate the driving risk of the overtaking process, we used the same training set but changed the model input to the subsets of the original test dataset, which corresponded to the four stages of the lane-changing process. The prediction results using the four subsets as the input are shown in Table 4. The results indicate that the most dangerous lane-changing stage was the intention stage, which was even more dangerous than the execution stage. The intention stage had the lowest probability of being safe, as well as the probability of transferring to a safe state and vice versa. This is probably because the drivers were more nervous when they were identifying the location of surrounding vehicles. When they began implementing the lane-changing behaviors, the decisions were already made without any doubt. Of course, the car following stage was the safest stage. Drivers were rarely in a risky condition. When they were overtaken by other vehicles, they might have felt dangerous. However, this condition would swiftly transition to the safe condition. Both intention stage and execution stage were more dangerous conditions than the overall probability. Of all the influence factors, the SDNN contributed most in the estimation of safety status, while fixation duration contributed most in the estimation of danger status. In this way, active safety protection measures may be considered in these stages in a reasonable way. 

## 6. Discussions

Based on the evaluations of influence factors, the fixation duration, saccade range, the SDNN of R–R interval, and the average speed were selected as the input of the HMM. However, some other performance indicators also had a significant influence on certain lane-changing stages. Some studies indicated that the frequency-domain HRV indicators have precise descriptions on the driver’s physiological status [42]. However, the comprehensive indicator LF/HF with the considerations of both LF and HF did not perform well in our research. The reason might be that both LF and HF have the same trend when the drivers are getting fatigued. The influence of individual frequency-domain HRV indicators will be tested in the future.

The results of the experiments indicate that the SDNN is suitable for estimating safety status, and the fixation duration is suitable for estimating danger status. The experimental results also indicate that the car following stage is the safest stage, and the intention stage and execution stage are relatively dangerous. In this way, the more general Bayesian network models may also be suitable to estimate the weight of each influence factor in the common car following stage or a specific lane-changing stage, which may improve the accuracy and efficiency of the proposed model.

Existing methods for estimating the driving risk are twofold: estimating the driving risk by evaluating the drivers’ physiological indicators or TTC. There are many physiological indicators to describe the driver’s state. However, it is difficult to distinguish which parameter has the best performance to estimate the driving risk. Some of these indicators may even have contradictory values in describing the same event. Thus, the difficulty of using the physiological indicators is the selection of the appropriate indicator and the establishment of the connection between the selected indicators and driving risk. The TTC is a frequently-used indicator to describe the driving risk. However, computing the TTC is not trivial, which usually be calculated based on the current or prior vehicle dynamic information. It is difficult to predict the driver’s behaviors in the near future, which may have a critical influence on the driving risk. A rough calculation of the driving risk based on TTC using our test data set found that 63 out of 75 predictions were consistent with the drivers’ perceptions. The results of verifying the 12 incorrect samples indicate that 9 drivers changed their operation maneuvers (like a sudden acceleration/deceleration), which lead to a significant change of the TTC. The rest 4 samples fell into the situation that is hard to distinguish the driving risk. Thus, the proposed method can locate the appropriate indicator to predict the driving risk, and combine all the possible indicators to predict the driving risk with reasonable accuracy.

The limitations of this research are listed as follows: ① the performance of the HMM is based on the training dataset. If the training set covers all possible patterns, the predictions are reliable. The HMM cannot process the data out of the range of the training data set; ② it is difficult to verify the proposed model in real time. One of the possible usages of the proposed model is proactive crash prevention. However, the performance of the proposed model can only be verified using post evaluation, which is based on a review of the driving process; ③ we only tested the driving risk prediction during the lane-changing process. More scenes of the driving process should be tested using the proposed method. 

## 7. Conclusions

In this study, an HMM-based model was proposed to predict the driving risk during the lane-changing process using the driver’s physiological data and vehicle dynamic data from related sensors. The following conclusions could be obtained based on the research process: 

(1) Sensors that can collect the driver’s physiological information and vehicle dynamic information can be utilized to predict the driving risk during the lane-changing process. Some of the eye movement data, the heart rate variability data, and the vehicle dynamic data were proven to have an influence on the driving risk. During the lane-changing process, the drivers’ HRV parameter became active firstly in the perception stage. Then, the eye movement became active in the intention stage. The implementation of operations can only be identified in the final execution stage, 

(2) A two-factor indicator analysis method was proposed to evaluate the influence of each parameter on the driving risk during the lane-changing process. The results indicate that the fixation duration, saccade range, the SDNN of R–R interval, and the average speed were proven to have a significant influence on the driving risk under different levels of service and driving duration.

(3) An HMM-based model was proposed to predict the driving risk during the lane-changing process. Based on the evaluation of the influence of eye movement, ECG, and vehicle dynamic data, the fixation duration, saccade range, SDNN, and average speed had significant influences on the driving risk of the lane-changing process and were selected as the observed parameters of the HMM. The proposed model was trained using 500 data samples from the lane-changing process of 25 drivers. The results indicated that 85.84% driving actions were safe and 14.16% driving actions were dangerous. The probability of maintaining a safe state was 90.67% and the probability of maintaining a dangerous state was 86.42%. The probabilities of transferring a safe state to a dangerous state and the opposite transferring process were 9.33% and 13.58%, respectively. The results matched the perceptions of the tested drivers with an accuracy rate of 90.67%.

(4) The proposed method can be utilized in the proactive crash prevention system with the related sensors and communication devices. Such sensors can be portable eye tracker, portable heart rate/ECG monitor, and OBD. With enough training data, the proposed method can estimate the probabilities of each driving state in the next few intervals. When the predicted value exceeds the threshold, the system can remind the driver, which will improve the driving safety condition. 

(5) Further research can focus on the mechanism of the interactions between the psychological indicators and TTC. With this information, the TTC can be adjusted considering the driver’s psychological status, which will make the predictions of driving risk given by TTC more accurate. Further discoveries on the relationship between physiological indicators and driving behaviors are also needed to acquire the driver’s decision-making mechanism.

## Figures and Tables

**Table 1:** The influence of eye movement indicators on driving risk. LOS—level of service.

Lane-Changing Stage	Influence Factors	LOS		Driving Duration	
		*I*_1_	*I*_2_	*I*_1_	*I*_2_
Car following	Fixation duration	4.966	0.073	1.952	0.101
	Saccade range	7.231	0.053	3.121	0.065
	Variability of pupil diameter	4.284	0.075	1.495	0.276
Perception	Fixation duration	5.759	0.065	1.959	0.103
	Saccade range	7.757	0.050	3.045	0.067
	Variability of pupil diameter	4.236	0.078	1.378	0.271
Intention	Fixation duration	78.899	<0.001	12.285	0.007
	Saccade range	52.443	0.003	6.591	0.029
	Variability of pupil diameter	6.267	0.050	1.938	0.264
Execution	Fixation duration	56.952	<0.001	13.646	0.006
	Saccade range	325.724	<0.001	8.960	0.004
	Variability of pupil diameter	5.938	0.052	1.267	0.3.03

**Table 2:** The influence of electrocardiogram (ECG) indicators on driving risk. LF/HF—ratio of absolute power of the low-frequency band to that of the high-frequency band; SDNN—standard deviation of normal to normal R–R intervals of the heart rate; CV—coefficient of variation of the R–R intervals.

Lane-Changing Stage	Influence Factors	LOS		Driving Duration	
		*I*_1_	*I*_2_	*I*_1_	*I*_2_
Car following	LF/HF	2.015	0.032	1.264	0.248
	SDNN	3.842	0.024	3.475	0.033
	CV	9.459	0.006	6.894	0.013
Perception	LF/HF	6.235	0.007	1.153	0.357
	SDNN	3.780	0.025	12.580	0.002
	CV	25.857	<0.001	8.237	0.003
Intention	LF/HF	7.822	0.007	3.265	0.135
	SDNN	4.330	0.016	15.710	0.002
	CV	49.166	<0.001	14.762	0.001
Execution	LF/HF	16.506	<0.001	13.208	0.033
	SDNN	4.370	0.015	9.515	0.001
	CV	74.259	<0.001	24.513	<0.001

**Table 3:** The influence of vehicle dynamic indicators on driving risk.

Lane-Changing Stage	Influence Factors	LOS		Driving Duration	
		*I*_1_	*I*_2_	*I*_1_	*I*_2_
Car following	Average speed	35,102.613	<0.001	50.673	<0.001
	Acceleration	1.521	<0.001	1.031	0.378
Perception	Average speed	929.482	<0.001	622.857	<0.001
	Acceleration	1.467	0.009	9.587	0.134
Intention	Average speed	2383.996	<0.001	727.462	<0.001
	Acceleration	4.383	0.013	2.064	0.103
Execution	Average speed	1491.669	<0.001	843.185	<0.001
	Acceleration	0.561	0.021	7.053	0.121

**Table 4:** The probability of the driving status of each lane-changing stage (unit: %).

	P_safe_	P_dangerous_	P_s-s_	P_s-d_	P_d-s_	P_d-d_
Car following	93.72	6.28	90.35	9.65	46.76	53.24
Perception	87.40	22.60	81.64	18.36	35.99	64.01
Intention	67.04	32.96	62.14	37.86	11.69	88.31
Execution	73.47	26.53	69.93	30.07	21.34	78.66
All	85.84	14.16	75.29	24.71	25.37	74.63
